# Fe-Doped Nickel Carbonate Hydroxide-Supported Ru Nanocluster Catalyst as Efficient OER Electrocatalysts

**DOI:** 10.3390/molecules30214209

**Published:** 2025-10-28

**Authors:** Qianqian Zhong, Jun Huang, Zhiyi Zeng, Xiaoqiang Wu, Jing He

**Affiliations:** 1 School of Biological Sciences, The University of Hong Kong, Hong Kong, China; 2 School of Mechanical Engineering, Chengdu University, Chengdu 610106, China; 3 School of Environment and Resource, Southwest University of Science and Technology, Mianyang 621010, China

**Keywords:** nickel carbonate hydroxide, ruthenium, electrodeposition, oxygen evolution reaction, electrocatalyst

## Abstract

The development of high-efficiency and stable oxygen evolution reaction (OER) electrocatalysts is crucial for sustainable hydrogen production via water splitting. Single-atom catalysts (SACs) represent a promising direction, yet their performance heavily relies on the support material. Herein, we report a highly active OER catalyst comprising ruthenium (Ru) species supported on Fe-doped nickel carbonate hydroxide (NFCH) grown on nickel foam (NF). The NFCH support, synthesized via a hydrothermal method, possesses a high specific surface area and excellent electrical conductivity. The incorporation of carbonate anions (CO_3_^2−^) enhances structural stability and interfacial hydrophilicity. Ru was subsequently decorated onto NFCH via electrodeposition to form the NFCH-Ru_x_ series (where x denotes the mmol amount of Ru precursor). The optimized NFCH-Ru_3_ catalyst exhibits outstanding OER performance in 1 M KOH, requiring a low overpotential of only 220 mV to achieve a current density of 10 mA cm^−2^, with a small Tafel slope of 40.92 mV dec^−1^. Furthermore, it demonstrates remarkable durability with negligible activity loss (2.9%) after 12 h of continuous operation, outperforming many recently reported non-precious metal-based catalysts. This work highlights the potential of metal carbonate hydroxides as superior supports for developing high-performance OER electrocatalysts.

## 1. Introduction

The escalating global energy demand and reliance on fossil fuels have led to severe environmental pollution, necessitating the exploration of new clean energy sources to support societal development [1,2,3]. Water electrolysis technology is widely recognized as a vital strategy for generating green hydrogen (H_2_) while reducing fossil fuel consumption [4,5,6]. The efficiency of water electrolysis hinges on the kinetics of the anodic OER and the cathodic hydrogen evolution reaction (HER), both heavily dependent on catalyst performance [7,8]. Currently, RuO_2_ and IrO_2_ are the state-of-the-art OER catalysts, but their widespread application is hindered by their scarcity and high cost [9,10]. Intensive research has been devoted to developing alternatives, including transition metal sulfides, phosphides, hydroxides, and oxides. However, matching the performance of RuO_2_ and IrO_2_ remains a significant challenge. SACs have garnered extensive attention due to their exceptional catalytic activity and nearly 100% metal atom utilization [11,12]. A critical challenge in SACs research is identifying suitable supports with high electrical conductivity and large specific surface area to stabilize the single atoms and facilitate electron/mass transfer [13,14].

Layered double hydroxide (LDH) is a two-dimensional material characterized by a high specific surface area, making it an ideal candidate for single-atom catalyst support [15]. However, LDH suffers from poor conductivity, and its structure can be unstable in alkaline media due to OH- intercalation, leading to dissolution in localized acidic environments. Furthermore, the brittleness of LDH can cause structural collapse when generated O_2_ bubbles cannot be effectively released, a limitation not even fully mitigated by advanced NiFe-LDH [16,17]. Carbonate hydroxide, with a similar layered structure, shares the advantages of high specific surface area but also the drawback of poor conductivity [18,19]. However, its distinct interlayer anion, CO_3_^2−^, provides higher OH- adsorption capacity, superior stability, and the ability to maintain structural charge balance compared to LDH. Additionally, CO_3_^2−^ enhances the wettability of the catalyst surface, improves electrolyte contact, and accelerates the reaction kinetics [20,21,22]. The conductivity issue can be mitigated by growing the material on a conductive nickel foam substrate.

The intrinsic activity of carbonate hydroxide can be improved by introducing other transition metals to modulate its electronic structure. For instance, Tang et al. found that Mn doping in CoCH adjusted the catalyst morphology, significantly increasing the electrochemically active surface area and exposing more active sites [23]. Karmakar et al. constructed nickel-substituted cobalt carbonate hydroxide with a unique hollow interchain morphology via a one-step reflux method, which facilitated electrode-electrolyte interaction [24]. Fe doping is particularly effective; it significantly enhances the OER activity of Ni^2+^-based catalysts by altering the local electronic environment and structure near the active sites of Ni(OH)_2_ [25,26]. Simultaneously, the incorporation of single atoms or nanoclusters into layered two-dimensional materials can maximize atomic utilization and increase the density of active sites [27,28]. The strong electron-metal support interaction can further modify the electronic structure of the catalyst, enhancing the adsorption capacity for OER intermediates [29,30,31].

Among various noble metal catalysts, ruthenium (Ru) has attracted considerable interest due to its relatively lower cost and optimal binding energy with reaction intermediates [32]. The introduction of Ru species can effectively modulate the electronic structure of the host catalyst, optimizing the binding strengths of OER intermediates (*O, *OH, *OOH) and thereby enhancing the overall catalytic efficiency [33,34,35]. For example, Wang et al. and Zhu et al. recently demonstrated the electronic modulation and exceptional stability achieved by incorporating Ru into NiFe-based catalysts [33,34]. Our work distinguishes itself by utilizing a Fe-doped carbonate hydroxide (NFCH) as the support, which we hypothesize offers superior stability compared to conventional LDHs.

Based on this rationale, we synthesized a series of Ru-decorated OER catalysts (NFCH-Ru_x_) by combining hydrothermal method with electrodeposition. The Fe-doped nickel carbonate hydroxide (NFCH) support itself exhibited performance comparable to NiFe-LDH (NF-LDH) with an overpotential of 260 mV at 10 mA cm^−2^ and demonstrated superior stability in a 12-h test. Subsequent electrodeposition of Ru onto NFCH yielded the NFCH-Ru_x_ catalysts, which showed substantial improvements in both activity and stability compared to NFCH and NF-LDH. The optimized NFCH-Ru_3_ catalyst achieved an exceptionally low overpotential of 220 mV at 10 mA cm^−2^, a Tafel slope of 40.79 mV dec^−1^, and minimal activity loss (2.9%) after 12 h of operation. These results not only provide an excellent candidate for OER electrocatalysis but also highlight carbonate hydroxide as a promising support material for future catalyst design.

## 2. Results and Discussion

### 2.1. Physical and Electrochemical Characterization of NF-LDH and NFCH

**Figure 1:**
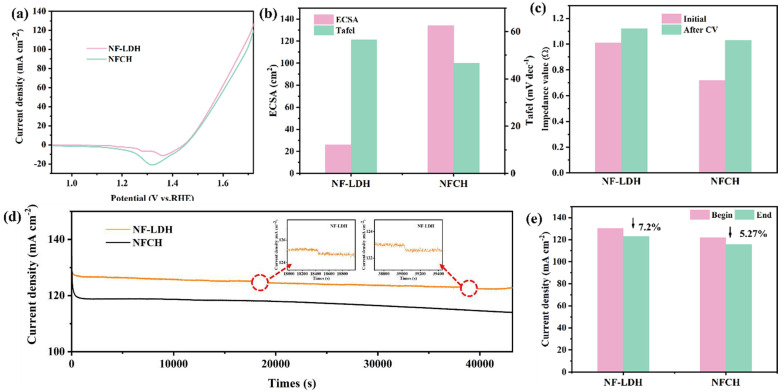
(**a**) LSV polarization curves of NF-LDH and NFCH. (**b**) Impedance value before and after CV cycle attenuation. (**c**) ECSA and Tafel slopes of NF-LDH and NFCH. (**d**) Long-term durability test of NF-LDH and NFCH at a potential of 1.724 V. (**e**) Comparison of current density attenuation before and after NF-LDH and NFCH long-term durability tests.

For clarity, the catalysts grown on nickel foam are hereafter referred to as NF-LDH and NFCH. The electrochemical performance of the synthesized NF-LDH and NFCH supports was first evaluated. Figure 1a shows the LSV polarization curves, revealing overpotentials of 252 mV for NF-LDH and 260 mV for NFCH at a current density of 10 mA cm^−2^, indicating comparable initial OER activity. Figure 1b presents the ECSA and Tafel slopes. NFCH exhibits a larger ECSA and a smaller Tafel slope than NF-LDH, suggesting a higher density of active sites and faster OER kinetics. These properties are beneficial for electrocatalytic performance and are desirable for a catalyst support [15]. The impedance plots and Tafel slopes are provided in Figure S1. Additionally, we evaluated the CV cycle durability of both catalysts. Figure S2 presents the CV polarization curves of both catalysts, while Figure 1c illustrates the impedance changes after 5000 CV cycles. Initially, NF-LDH exhibited lower impedance. However, after cycling, the impedance of NFCH increased less significantly than that of NF-LDH, indicating better retention of conductivity. The long-term durability was assessed by CA tests at 1.724 V. As shown in Figure 1d, the platform current density of NF-LDH showed a significant decline, while NFCH maintained a more stable current. Figure 1e quantifies the current density attenuation after 12 h, showing a 7.2% loss for NF-LDH compared to only 5.27% for NFCH. These results underscore NFCH’s excellent stability over extended working periods, confirming its suitability as an effective catalyst support.

**Figure 2:**
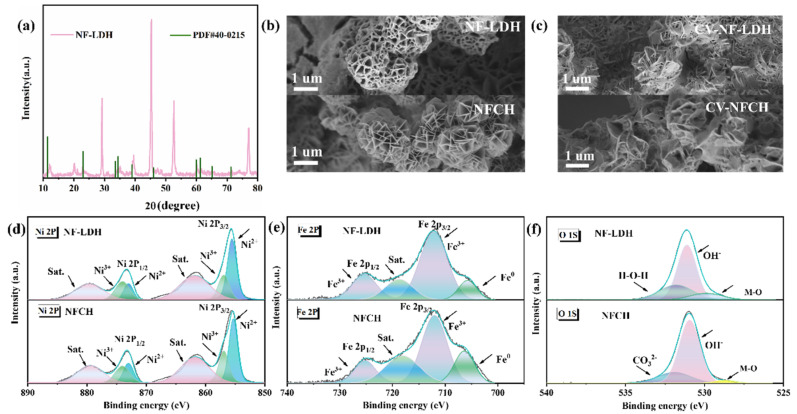
(**a**) XRD patterns of NF-LDH. (**b**) Initial morphology of NF-LDH and NFCH. (**c**) Morphology of NF-LDH and NFCH after CV attenuation test. (**d**–**f**) XPS analysis of NF-LDH and NFCH.

To further demonstrate the superior properties of NFCH as a catalyst support compared to NF-LDH, we conducted a detailed analysis of their microstructures and elemental valence states, as shown in Figure 2. Figure 2a presents the XRD pattern of NF-LDH. The diffraction peaks match well with the standard pattern for NiFe-LDH (PDF#40-0215) [35], confirming the successful synthesis of NF-LDH on the nickel foam substrate. Figure 2b,c show the SEM images of NF-LDH and NFCH before and after the CV cycle attenuation test, respectively. Both materials initially exhibit spherical morphologies composed of stacked lamellae. After cycling, NFCH shows more pronounced morphological changes, consistent with the impedance change in Figure 1c, but the overall spherical structure remains intact (Figure S3), underpinning its long-term stability. This indicates that NFCH retains better performance after long-term stability testing. Previous studies have demonstrated that Ni^3+^ is more effective than Ni^2+^ as an active site for the OER, and the content of Ni^3+^ significantly influences OER activity. High-valence species as catalytic active sites can optimize the adsorption energy of OH- and promote the deprotonation of -OOH to produce oxygen [36,37,38]. XPS analysis was conducted to investigate the surface chemical states. The Ni 2p spectra (Figure 2d) show two spin–orbit doublets corresponding to Ni 2p_3/2_ and Ni 2p_1/2_. The peaks at binding energies of 856.9 eV and 874.0 eV are assigned to Ni^3+^ [39]. The similar Ni^3+^ content in NF-LDH and NFCH correlates with their comparable initial OER activities. The Fe 2p spectra (Figure 2e) confirm the presence of Fe^3+^ in both catalysts. The O 1s spectrum (Figure 2f) of NFCH reveals a distinct component at 531.5 eV, attributed to CO_3_^2−^ species [40,41], which is absent in NF-LDH. The presence of CO_3_^2−^ not only verifies the formation of carbonate hydroxide but also contributes to the enhanced hydrophilicity and structural stability of NFCH, explaining its superior long-term durability. For more detailed XPS analysis, see Figure S4.

### 2.2. Physical and Electrochemical Characterization of NFCH-Ru

**Figure 3:**
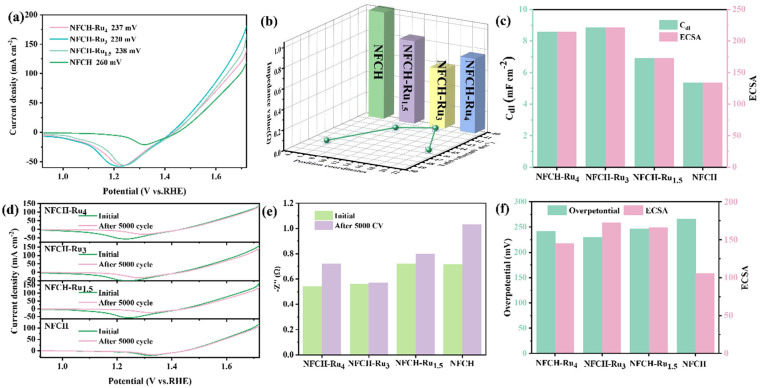
(**a**) LSV polarization curve of NFCH-Ru_x_. (**b**) Impedance value and Tafel slope 3d plot of NFCH-Ru_x_. (**c**) C_dl_ value and ECSA value of NFCH-Ru_x_. (**d**) The initial state and the LSV polarization curve after CV cycle attenuation of NFCH-Ru_x_. (**e**) Impedance value before and after CV attenuation of NFCH-Ru_x_. (**f**) Relationship between overpotential and ECSA of NFCH-Ru_x_.

After identifying NFCH as a promising support, we decorated it with Ru via electrodeposition to form the NFCH-Ru_x_ series (x = 4, 3, 1.5 mmol Ru precursor). Figure 3a shows the LSV curves. The deposition of Ru significantly enhanced OER activity, with NFCH-Ru_3_ showing the lowest overpotential (220 mV at 10 mA cm^−2^). This confirms the excellent catalytic activity of the supported Ru nanoclusters. Figure 3b compares the Tafel slopes and impedance. NFCH-Ru_3_ exhibits the smallest Tafel slope (40.92 mV dec^−1^) and the lowest impedance, indicating favorable kinetics and efficient charge transfer. As shown in Figure S5, NFCH-Ru_3_ also compares favorably with other state-of-the-art OER catalysts currently under study, demonstrating superior catalytic performance in terms of both overpotential and Tafel slope, placing it among the best overall OER catalysts. The electrochemically active surface area (ECSA) is an important parameter for evaluating catalyst quality. Figure 3c presents the C_dl_ and corresponding ECSA values. NFCH-Ru_3_ has the largest ECSA (237 cm^2^), consistent with its superior performance. To assess CV cycle durability, all Ru-deposited catalysts underwent CV cycle attenuation tests. Figure 3d shows the LSV curves before and after 5000 CV cycles. The overpotential of NFCH-Ru_3_ increased only marginally. Figure 3e shows the impedance changes after cycling; NFCH-Ru_3_ maintained the lowest and most stable impedance. This stability is attributed to NFCH’s good electrical conductivity and Ru’s enhancement of electron transfer rate. Finally, Figure 3f comparatively presents the overpotential and ECSA values after the CV test, confirming that NFCH-Ru_3_ retains the best overall characteristics.

**Figure 4:**
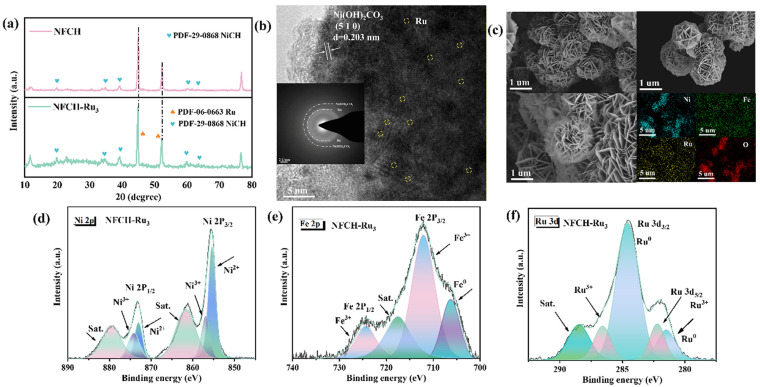
(**a**) XRD pattern. (**b**) TEM image. (**c**) TEM image and element mapping. (**d**–**f**) XPS analysis.

We conducted a series of characterization methods to investigate the microstructure, valence states, and electronic structure of NFCH-Ru_3_, aiming to elucidate the reasons for its remarkable performance and CV durability under Ru nanoclusters loading. To verify the successful incorporation of Ru nanoclusters onto the NFCH substrate, we employed XRD and TEM analyses. The XRD patterns of NFCH and NFCH-Ru_3_ (Figure 4a) both match the standard pattern for nickel carbonate hydroxide (NiCH, PDF#29-0868). The characteristic peaks at 2θ = 12.1°, 24.6°, 33.5°, and 59.8° can be indexed to the (110), (300), (021), and (060) planes of NiCH, respectively. A slight left-shift of the NF peaks (e.g., from 44.95° to 44.88°) in NFCH-Ru_3_ suggests lattice strain, possibly induced by the incorporation of Ru species [39]. No distinct peaks for crystalline Ru phases were detected, which, while not confirming atomic dispersion, suggests the absence of large Ru nanoparticles. The lattice fringes and spacing of NiCH were analyzed using TEM images combined with Figure 4b, which align with the standard lattice spacing in the PDF card, directly confirming the successful synthesis of NiCH. TEM images (Figure 4b) reveal the layered structure of NFCH. Lattice fringes with a spacing of 0.26 nm correspond to the (300) plane of NiCH. Elemental mapping (Figure 4c) shows a uniform distribution of Ni, Fe, O, C, and Ru, confirming the successful and homogeneous incorporation of Ru species into the NFCH matrix [32]. XPS analysis (Figure 4d–f) was performed to probe the electronic structure. The Ni and Fe spectra in NFCH-Ru_3_ are similar to those in NFCH. However, the Ni^3+^/Ni^2+^ ratio is higher in NFCH-Ru_3_, which may contribute to its enhanced activity. The Ru 3d spectrum (Figure 4f) shows peaks at 280.8 eV and 285.0 eV, assigned to Ru^0^ 3d_5/2_ and Ru^3+^ 3d_3/2_, respectively [32,40], indicating the presence of both metallic and oxidized Ru species. The electronic interaction between Ru and the NFCH support is a key factor for the improved OER performance.

**Figure 5:**
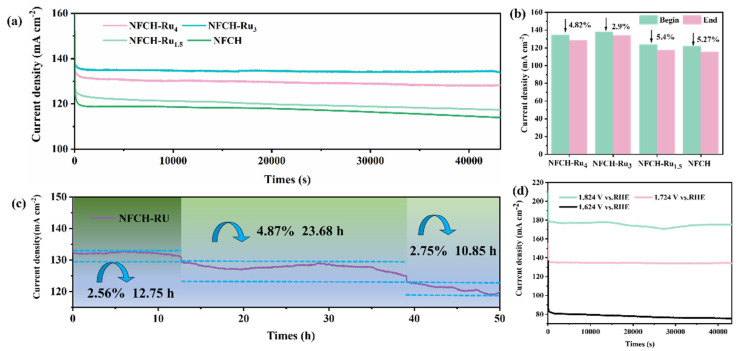
(**a**) Long-term durability test of NFCH and NFCH-Ru_x_ at a voltage of 1.724 V. (**b**) Current density attenuation rate of each sample after 12-h durability test. (**c**) I-T chart of NFCH-Ru_3_ after 50 h of durability testing. (**d**) Durability testing of NFCH at different voltages.

To evaluate the stability of NFCH-Ru_x_ in an alkaline environment, we employed the chronoamperometry (CA) method to assess its performance in a 1 M KOH solution. As shown in Figure 5a, NFCH-Ru_3_ exhibits superior catalytic performance among the NFCH-Ru_x_ series, maintaining the highest output platform current at the same test voltage. The I-T curves for each sample fully conform to their respective performance trends, and due to the stabilizing effect of CO_3_^2−^, these curves remain stable with no significant attenuation, intuitively demonstrating that carbonate hydroxide has distinct advantages over LDH in terms of stability.

Figure 5b shows the current density decay after 12 h; NFCH-Ru_3_ had the smallest decay rate (2.9%). To further validate this conclusion, CA tests were extended up to 50 h. As shown in Figure 5c, within the first 12 h, the current density of NFCH-Ru_3_ attenuated by 2.56%. After 23.6 h, the performance attenuated again, with the current density decreasing by 4.87%. However, during this period, there was some recovery in current density, likely due to internal structural reconstruction, leading to a self-adaptive reactivation of performance. In the final 10.85 h of testing, the current density decreased by 2.75%. After 50 h of CA testing, the total activity attenuation was only 7.34%, comparable to the attenuation observed in NF-LDH after 12 h of testing, thus confirming the excellent long-term stability of NFCH-Ru_3_. Additionally, we investigated the stability of NFCH-Ru_3_ under different voltage conditions, as shown in Figure 5d. It is evident that the stability of NFCH-Ru_3_ is compromised under both high and low voltage conditions, particularly under high voltage, where the I-T curve exhibits significant fluctuations. This indicates that NFCH-Ru_3_ is less resistant to high voltages. Conversely, under low voltage conditions, the I-T curve remains relatively stable, with greater current density attenuation observed at higher voltages compared to 1.724 V_RHE_.

**Figure 6:**
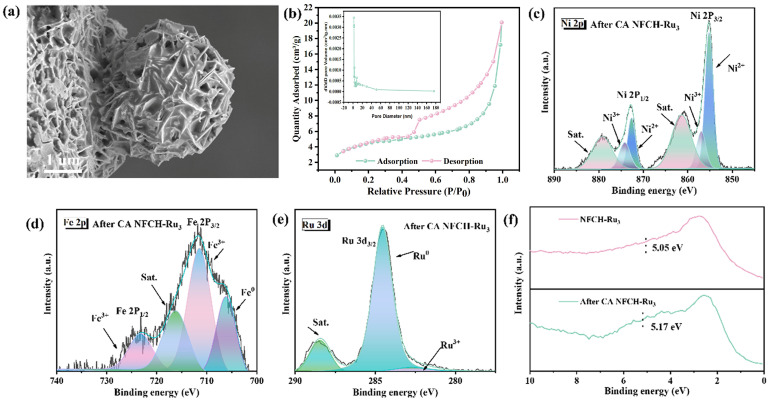
(**a**) SEM image of NFCH-Ru_3_ after CA test. (**b**) BET of NFCH-Ru_3_. (**c**–**e**) XPS analysis of NFCH-Ru_3_ after CA test. (**f**) D-band center of NFCH-Ru_3_.

To understand the reasons behind the exceptional stability of NFCH-Ru_3_, we analyzed its morphology, valence state, and electronic structure following the chronoamperometry (CA) test. Firstly, as illustrated in Figure 6a, SEM shows the spherical morphology was largely preserved after 12 h of CA testing. This is in stark contrast to the severe structural damage observed for NF-LDH and NFCH under the same conditions, particularly the near-complete structural failure of NF-LDH. The preservation of NFCH-Ru_3_’s structural integrity highlights its superior stability. Secondly, we investigated the specific surface area of NFCH-Ru_3_ in its initial state. As shown in Figure 6b, BET analysis indicates a type II isotherm with an H4 hysteresis loop, characteristic of mesoporous materials. The calculated specific surface area is 98.5 m^2^/g, which facilitates the exposure of active sites. This not only enhances catalytic activity but also contributes to improved stability. According to general catalyst characteristics, a larger specific surface area and pore size typically lead to greater exposure of active sites and enhanced adsorption of active molecules, facilitating rapid migration, enhancing electron transfer, and improving OER performance [41]. XPS analysis after the CA test (Figure 6c–e) shows a decrease in Ni and Ru content, suggesting consumption of surface species during OER. The Ru 3d spectrum shows a significant reduction in the Ru^3+^ signal, indicating its involvement in the reaction. Figure 6c shows the valence distribution of Ni elements in NFCH-Ru_3_ after the reaction. It is evident that the content of Ni^2+^ and Ni^3+^ has decreased compared to before the reaction, likely due to the conversion of Ni^2+^ to Ni^3+^ during the reaction process and the subsequent loss of Ni^3+^ as the primary reactive site. The valence state of Fe, as shown in Figure 6d, remains consistent with pre-reaction conditions, although its content has reduced, which is a normal phenomenon post-reaction. The D-band center analysis (Figure 6f) shows a slight upshift after the reaction, bringing it closer to the Fermi level, which is generally associated with optimized intermediate adsorption and improved OER activity. However, the XPS test results for Ru element exhibit significant changes. The Ru^3+^ peak at the Ru 3d_3/2_ position was completely consumed, and the overall content of Ru decreased. The Ru element at the Ru 3d_5/2_ position appears to be fully reactive. This suggests that Ru nanoclusters are consumed as active sites, while Ru^3+^ at the Ru 3d_3/2_ position may be reduced to elemental Ru during the reaction, serving as active sites. Finally, Figure 4d analyzes the D-band center position before and after the catalyst reaction. After the reaction, the D-band center shifted 0.12 eV to the right, aligning with the principle that catalysts closer to the original Fermi level exhibit better performance.

In summary, the OER activity of NFCH-Ru_3_ is among the best-performing catalysts reported recently. For instance, its overpotential of 220 mV at 10 mA cm^−2^ and Tafel slope of 40.92 mV dec^−1^ are superior to those of many Ru-modified catalysts, such as Ru-NiFe LDH and other state-of-the-art non-precious metal catalysts. This exceptional performance can be attributed to the synergistic combination of the high-surface-area, stable NFCH support and the highly active, dispersed Ru species, which collectively enhance charge transfer and optimize the adsorption of reaction intermediates.

## 3. Experimental Section

### 3.1. Materials

Nickel chloride hexahydrate (NiCl_2_⋅6H_2_O, 99.9%, Chengdu Kelong Chemical Reagent Factory, Chengdu, China); Iron nitrate nonahydrate (Fe(NO_3_)_3_⋅9H_2_O, 99.9%, Shanghai Macklin Biochemical Technology Co., Ltd., Shanghai, China); Nickel nitrate hexahydrate (Ni(NO_3_)_2_⋅6H_2_O, 99.9%, Tianjin Damao chemical reagent production, Tianjin, China); Ruthenium (III) chloride (RuCl_3_, 99.5%, Shanghai Titan Technology Co., Ltd., Shanghai, China); Potassium hydroxide (KOH, 99.9%, Chengdu Kelong Chemical Reagent Factory); Urea (NH_2_CONH_2_, 99.9%, Shanghai Titan Technology Co., Ltd.); Ammonium fluoride (NH_4_F, 99.9%, Shanghai Titan Technology Co., Ltd.). The deionized water’s electrical resistivity is higher than 18 MΩ cm^−1^. All chemicals are used directly without further purification, and the deionized water comes from the deionized water purification system in our laboratory and is used in all experiments.

### 3.2. Preparation of NF-LDH

Nickel foam (NF) was cut into 2 cm × 2 cm pieces and ultrasonically cleaned in hydrochloric acid, ethanol, acetone, and deionized water successively for 5 min each. After cleaning, all samples were dried in a blast oven. A precursor solution for NF-LDH was prepared by dissolving 0.138 g of Fe(NO_3_)_3_⋅9H_2_O, 0.15 g of NH_4_F, 0.11 g of NiCl_2_⋅6H_2_O, and 0.6 g of urea in 20 mL of deionized water under stirring. The cleaned NF piece and the precursor solution were transferred into a 100 mL Teflon-lined autoclave, which was sealed and maintained at 120 °C for 12 h. After the reaction, the autoclave was cooled to room temperature naturally. The resulting NF-LDH sample was removed, thoroughly rinsed with deionized water and ethanol, and then dried at 60 °C for 6 h in a vacuum oven.

### 3.3. Preparation of NFCH

The preparation of NFCH followed a similar procedure to NF-LDH. Specifically, 0.58 g Fe(NO_3_)_3_⋅4H_2_O, 0.054 g NH_4_F, 0.814 g Ni(NO_3_)_2_⋅6H_2_O, and 0.216 g urea were dissolved in 30 mL deionized water to form the NFCH precursor solution. The NF piece and the solution were then treated in a 100 mL autoclave at 170 °C for 8 h. The obtained NFCH was cleaned and dried following the same procedure as for NF-LDH.

### 3.4. Preparation of NFCH-Ru_x_

**Figure 7:**
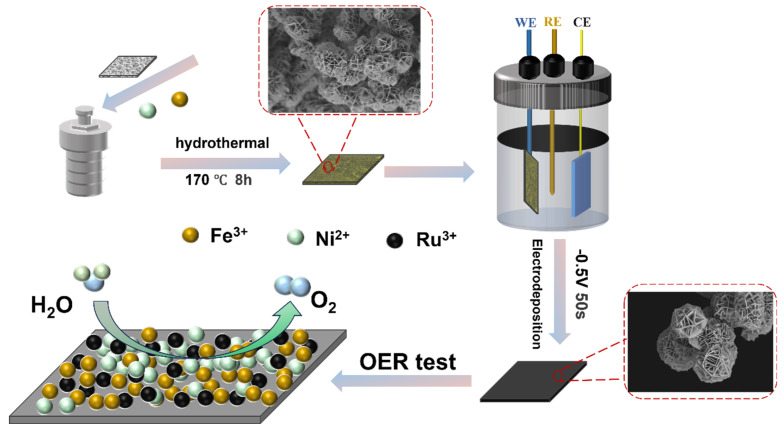
Schematic diagram of the NFCH-Ru_x_ preparation process.

NFCH-Ru_x_ was prepared via an electrodeposition method. Briefly, x mmol (x = 4, 3, 1.5) of RuCl_3_ was dissolved in 20 mL of deionized water under stirring to form a Ru precursor solution. A dried NFCH/NF sample (1 cm × 2 cm) was immersed in the Ru precursor solution and served as the working electrode. Electrodeposition was carried out at a constant voltage of −0.5 V (vs. Ag/AgCl) for 50 s. The obtained NFCH-Ru_x_ was then dried in a vacuum oven at 60 °C for 6 h. Figure 7 presents a schematic diagram of the NFCH-Ru_x_ preparation process.

### 3.5. Physical Characterization

To observe the morphology and structure of the samples, we employed a scanning electron microscope (SEM, ZEISS GeminiSEM 300, Jena, Germany) and a transmission electron microscope (TEM, FEI Tecnai G2 F20, Chapel Hill, NC, USA). Additionally, the composition of elements and chemical bonds on the sample surface was analyzed using an EDS and X-ray photoelectron spectrometer (XPS, Thermo Scientific K-Alpha, Waltham, MA, USA), with all spectra calibrated using the C1s peak at 284.5 eV. Furthermore, a fully automatic surface area and porosity analyzer (Micromeritics ASAP 2460, Norcross, GA, USA) was utilized to assess the specific surface area and pore size of the sample. Finally, the sample was studied using an X-ray diffractometer (XRD, Ultima IV, Rigaku, Tokyo, Japan) under the following conditions: 40 KV tube voltage, 10–80° scanning angle, 0-06 °s^−1^ stepping angle, and 1 s interval sampling time.

### 3.6. Electrochemical Measurements

All electrochemical tests were performed at room temperature using a CHI760E electrochemical workstation (Shanghai Chenhua Instrument Co., Ltd., Shanghai, China) in a standard three-electrode system with Hg/HgO as the reference electrode, a platinum wire as the counter electrode, and the catalyst on NF as the working electrode. The OER performance was evaluated in 1 M KOH using linear sweep voltammetry (LSV), cyclic voltammetry (CV), and chronoamperometry (CA). All potentials were converted to the reversible hydrogen electrode (RHE) scale using the equation E_RHE_ = E + E_Hg/HgO_ + 0.0591*(pH). Before formal testing, the catalyst was activated by 100 CV cycles at a scan rate of 100 mV/s between 0 and 0.8 V (vs. Hg/HgO). LSV was conducted at a scan rate of 10 mV/s. Electrochemical impedance spectroscopy (EIS) was measured from 0.1 Hz to 100 kHz at a bias potential of 0.75 V (vs. RHE) with an amplitude of 10 mV. The electrochemical active surface area (ECSA) was estimated from the double-layer capacitance (C_dl_), which was determined by CV measurements in a non-faradaic potential region at scan rates of 20, 40, 60, 80, and 100 mV/s. The ECSA was calculated using the formula ECSA = C_dl_/C_s_, where C_s_ is the specific capacitance of a flat standard, typically taken as 0.040 mF cm^−2^ in alkaline electrolyte [41]. The CV cycle durability test involved 5000 cycles at a scan rate of 100 mV/s. CA tests were conducted to assess stability.

## 4. Conclusions

In summary, we successfully developed a high-performance OER electrocatalyst by decorating Ru species onto a Fe-doped nickel carbonate hydroxide (NFCH) support. The NFCH carrier itself demonstrated comparable activity and superior long-term stability compared to traditional NiFe-LDH, attributed to the stabilizing effect of CO_3_^2−^ anions. The optimized NFCH-Ru_3_ catalyst exhibited exceptional OER performance with an ultralow overpotential of 220 mV at 10 mA cm^−2^, a small Tafel slope of 40.92 mV dec^−1^, and outstanding stability (only 2.9% activity loss after 12 h). The enhancement is attributed to the synergistic combination of a high-surface-area, stable NFCH support and highly active Ru species, which collectively increase the number of active sites and enhance the overall electrocatalytic activity. This work underscores the great potential of transition metal carbonate hydroxides as robust supports for designing efficient and durable electrocatalysts for energy conversion applications.

## Data Availability

The original contributions presented in this study are included in the article/Supplementary Materials. Further inquiries can be directed to the corresponding authors.

## Supplementary Materials

The following supporting information can be downloaded at https://www.mdpi.com/article/10.3390/molecules30214209/s1, Figure S1: (a) Tafel value of NF-LDH. (b) Tafel value of NFCH-Rux. (c) EIS of NF-DLH. (d) EIS of NFCH. Figure S2: (a,b) CV curves of all samples. (c,d) CV curves of all samples after CV cycle attenuation test. Figure S3: (a) SEM images of CV-NFCH. (b) SEM images of NF-LDH. (c) SEM images of CV-NFCH-Ru_3_. (d) SEM images of CA-NFCH-Ru_3_. Figure S4: (a) XPS analysis of NF-LDH and NFCH. (b) The D-band center of NF-LDH and NFCH. (c) Survey spectrum. Figure S5: Comparison of overpotential and Tafel slope of different catalysts. Refs. [42,43,44,45,46,47,48,49,50,51,52,53,54,55,56,57,58,59,60] are cited in the Supplementary Materials.
