# Supplementary material for: Fe-Doped Nickel Carbonate Hydroxide-Supported Ru Nanocluster Catalyst as Efficient OER Electrocatalysts
Source: Molecules. 2025 Oct 28;30(21):4209. doi: 10.3390/molecules30214209 (PMC12609940; doi:10.3390/molecules30214209)
Supplement: Supplementary file 1 [file molecules-30-04209-s001.zip › molecules-3878917-supplementary.pdf]

Supporting Information

for

Fe-Doped Nickel Carbonate Hydroxide-Supported Ru Nanocluster Catalyst as Efficient OER Electrocatalysts

Qianqian Zhong [1,†], Jun Huang [2,†], Zhiyi Zeng [2], Xiaoqiang Wu [2,*] and Jing He [3,*]

[1] School of Biological Sciences, The University of Hong Kong, Hong Kong, China
[2] School of Mechanical Engineering, Chengdu University, Chengdu 610106, China
[3] School of Environment and Resource, Southwest University of Science and Technology, Mianyang 621010, China
* Correspondence: wuxiaoqiang@cdu.edu.cn (X.W.); hejing@swust.edu.cn (J.H.)
† These authors contributed equally to this work

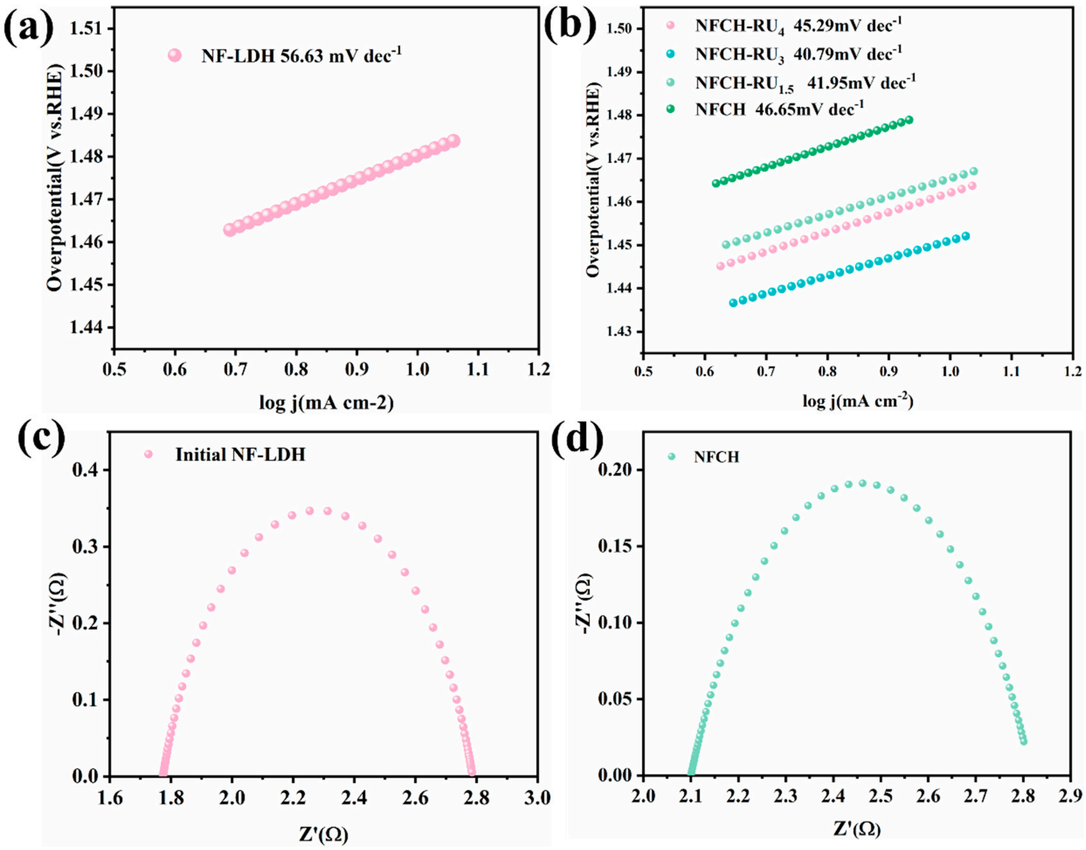

Fig. S1. (a) Tafel value of NF-LDH. (b) Tafel value of NFCH-Ru$_x$. (c) EIS of NF-DLH. (d) EIS of NFCH.

Fig. S1 (a) and (b) are the Tafell slope diagrams of the catalysts involved in this paper. Tafel is an intuitive representation of the reaction kinetics of OER catalysts. Generally, the smaller the value, the faster the catalytic rate. (c) and (d) are the EIS spectra of NF-LDH and NFCH, and the charge transfer resistance (Rct) of the catalyst is expressed by the semicircular diameter of the Nyquist curve.[1]

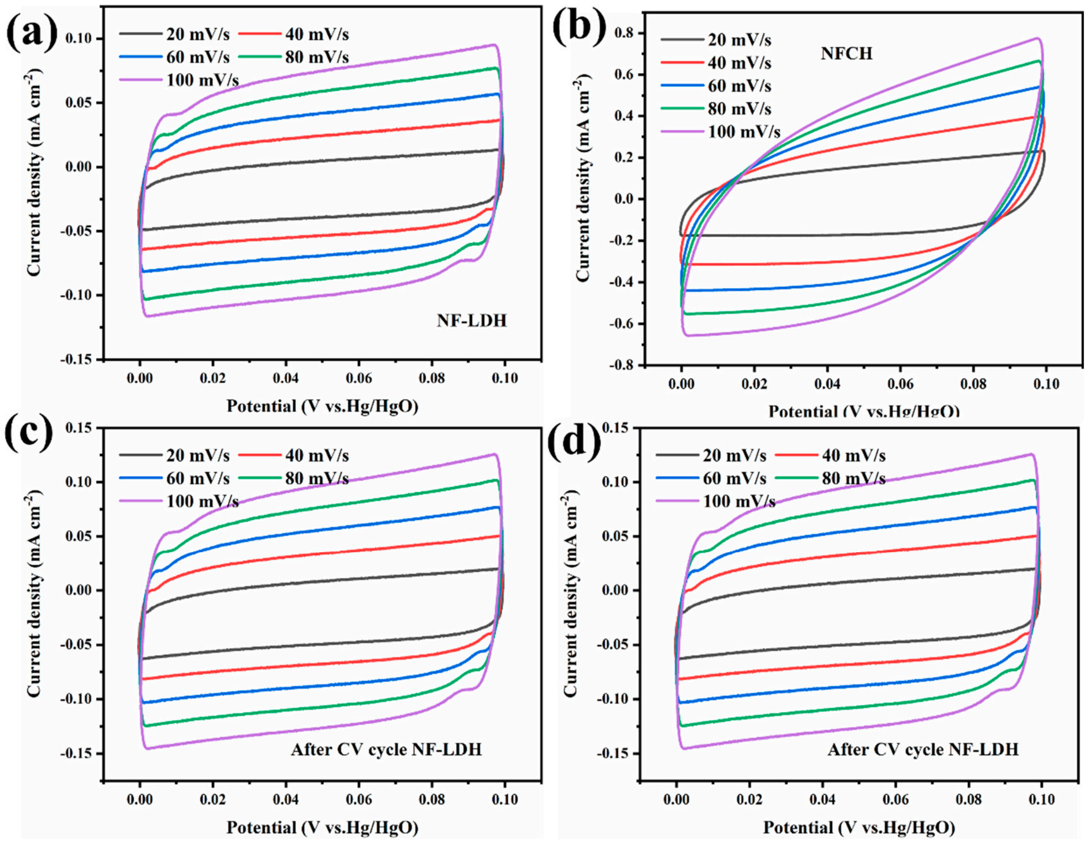

Fig. S2. (a)-(b) CV curves of all samples. (c)-(d) CV curves of all samples after CV cycle attenuation test.

Fig. S2 is CV curves of all samples before and after CV cycle attenuation, which is an important basis for calculating ECSA in this paper.[2] ECSA can be calculated according to the **Formula S1**,

$$\text{ECSA}={}^{C_{dl}}/_{C_s} \qquad \text{(S1)}$$

C_s is usually valued between 0.022 and 0.130 mF cm^{-2}.[3]

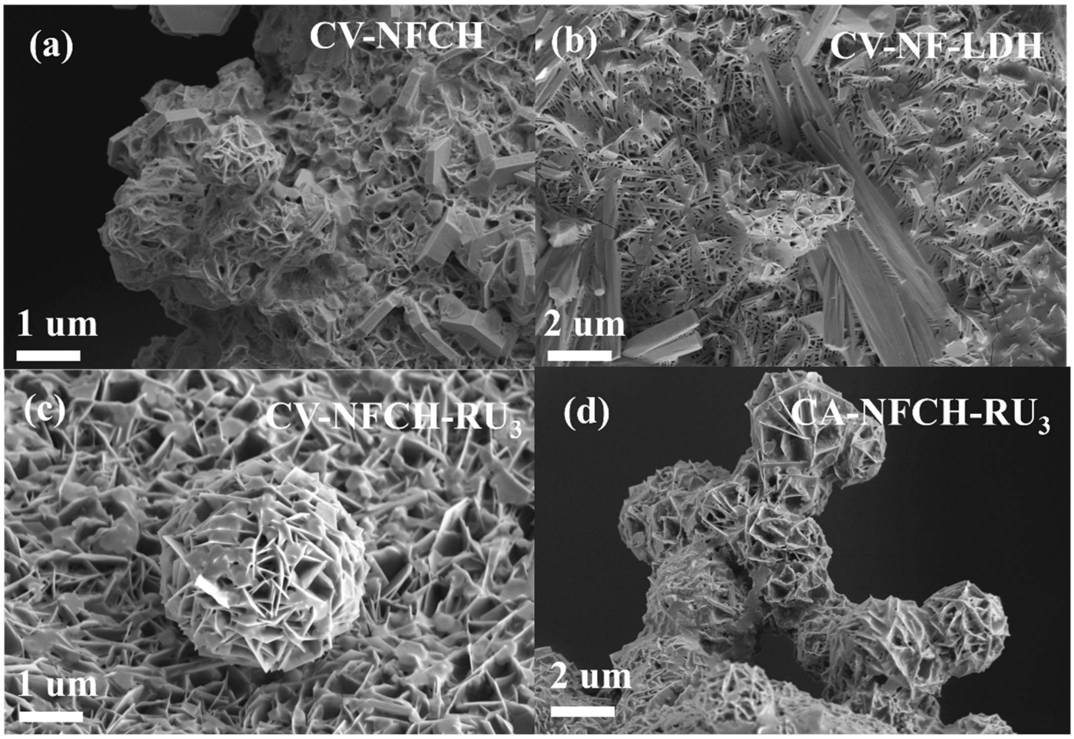

Fig. S3. (a) SEM images of CV-NFCH. (b) SEM images of NF-LDH. (c) SEM images of CV-NFCH-Ru_3. (d) SEM images of CA-NFCH-Ru_3.

Fig. S3 shows the SEM images of each sample after different reaction stages. After the same CV cyclic attenuation test, it can be seen from the process that the spherical morphology of NF-LDH and NFCH is not intact, while the morphology of NFCH-Ru remains in a complete spherical shape and the lamella-structure is still clearly visible. All these signs indicate that the introduction of NFCH-Ru_3 Ru Single atom not only increases the activity of NFCH, but also further improves its stability. Finally, after 12 hours of CA test, NFCH-Ru still maintains a spherical structure, which further explains its excellent stability.

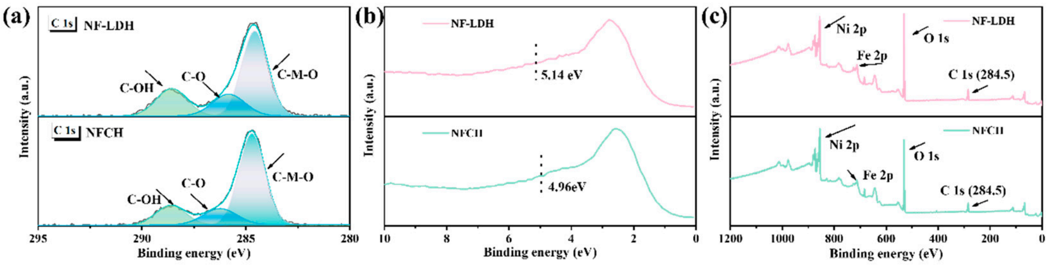

Fig. S4. (a) XPS analysis of NF-LDH and NFCH. (b) The D-band center of NF-LDH and NFCH. (c) Survey spectrum.

The D-band center can explain the binding strength of the active site and the reaction intermediate on the catalyst. When the binding of the active site and the reaction intermediate is closer, it is more conducive to the enhancement of OER activity.[4–6]

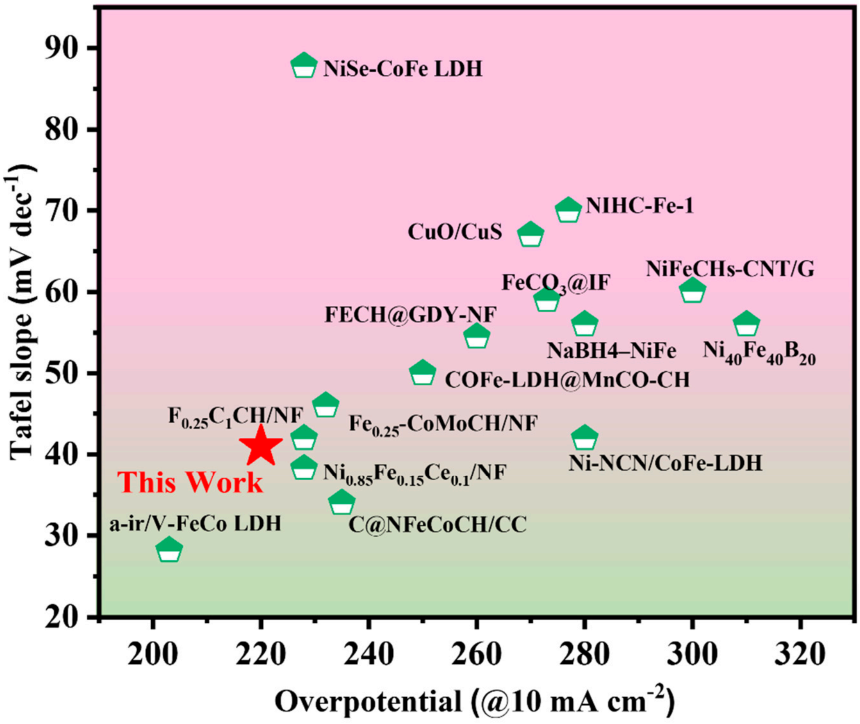

Fig. S5. Comparison of overpotential and Tafel slope of different catalysts[7–14,14–20].

References

[1] W. Hu, Q. Liu, T. Lv, F. Zhou, Y. Zhong, Impact of interfacial CoOOH on OER catalytic activities and electrochemical behaviors of bimetallic CoxNi-LDH nanosheet catalysts, Electrochimica Acta 381 (2021) 138276. https://doi.org/10.1016/j.electacta.2021.138276.

[2] F. Urbain, V. Smirnov, J.-P. Becker, A. Lambertz, U. Rau, F. Finger, Light-induced degradation of adapted quadruple junction thin film silicon solar cells for photoelectrochemical water splitting, Solar Energy Materials and Solar Cells 145 (2016) 142–147. https://doi.org/10.1016/j.solmat.2015.07.033.

[3] C.C.L. McCrory, S. Jung, J.C. Peters, T.F. Jaramillo, Benchmarking Heterogeneous Electrocatalysts for the Oxygen Evolution Reaction, J. Am. Chem. Soc. 135 (2013) 16977–16987. https://doi.org/10.1021/ja407115p.

[4] H. Lee, O. Gwon, K. Choi, L. Zhang, J. Zhou, J. Park, J.-W. Yoo, J.-Q. Wang, J.H. Lee, G. Kim, Enhancing Bifunctional Electrocatalytic Activities via Metal d-Band Center Lift Induced by Oxygen Vacancy on the Subsurface of Perovskites, ACS Catal. 10 (2020) 4664–4670. https://doi.org/10.1021/acscatal.0c01104.

[5] P. Zhang, S. Liu, J. Zhou, L. Zhou, B. Li, S. Li, X. Wu, Y. Chen, X. Li, X. Sheng, Y. Liu, J. Jiang, Co-Adjusting d-Band Center of Fe to Accelerate Proton Coupling for Efficient Oxygen Electrocatalysis, Small (2023) 2307662. https://doi.org/10.1002/smll.202307662.

[6] D. Luo, B. Yang, Z. Mei, Q. Kang, G. Chen, X. Liu, N. Zhang, Tuning the d-Band States of Ni-Based Serpentine Materials via Fe^{3+} Doping for Efficient Oxygen Evolution Reaction, ACS Appl. Mater. Interfaces 14 (2022) 52857–52867. https://doi.org/10.1021/acsami.2c14720.

[7] L. Hui, D. Jia, H. Yu, Y. Xue, Y. Li, Ultrathin Graphdiyne-Wrapped Iron Carbonate Hydroxide Nanosheets toward Efficient Water Splitting, ACS Appl. Mater. Interfaces 11 (2019) 2618–2625. https://doi.org/10.1021/acsami.8b01887.

[8] X. Deng, J. Chen, Q. Chen, Y. Zhou, X. Liu, J. Zhang, G. Wang, R. Wang, The synergistic effect of Ir and oxygen vacancies on Enhancing the OER performance of Surface-Reconstructed FeCo LDH, Applied Surface Science 665 (2024) 160310. https://doi.org/10.1016/j.apsusc.2024.160310.

[9] M. Cai, X. Lu, Z. Zou, K. Guo, P. Xi, C. Xu, The Energy Level Regulation of CoMo Carbonate Hydroxide for the Enhanced Oxygen Evolution Reaction Activity, (n.d.).

[10] W. Zhu, G. Zhu, J. Hu, Y. Zhu, H. Chen, C. Yao, Z. Pi, S. Zhu, E. Li, Poorly crystallized nickel hydroxide carbonate loading with Fe3+ ions as improved electrocatalysts for oxygen evolution, Inorganic Chemistry Communications 114 (2020) 107851. https://doi.org/10.1016/j.inoche.2020.107851.

[11] G. Ma, M. Qin, W. Tan, Z. Fan, X. Xin, Optimizing OER performance with CoFe-LDH@MnCo–CH nanoneedle arrays: Leveraging p-n junctions in electrocatalysis, International Journal of Hydrogen Energy 60 (2024) 902–908. https://doi.org/10.1016/j.ijhydene.2024.01.363.

[12] Y. Wang, S. Tao, H. Lin, S. Han, W. Zhong, Y. Xie, J. Hu, S. Yang, $NaBH_4$ induces

a high ratio of Ni^{3+} /Ni^{2+} boosting OER activity of the NiFe LDH electrocatalyst, RSC Adv. 10 (2020) 33475–33482. https://doi.org/10.1039/D0RA06617F.

[13] L. Hui, Y. Xue, D. Jia, H. Yu, C. Zhang, Y. Li, Multifunctional Single-Crystallized Carbonate Hydroxides as Highly Efficient Electrocatalyst for Full Water splitting, Advanced Energy Materials 8 (2018) 1800175. https://doi.org/10.1002/aenm.201800175.

[14] Z. Hou, F. Fan, Z. Wang, Y. Deng, Y. Du, Heterostructural NiSe-CoFe LDH as a highly effective and stable electrocatalyst for the oxygen evolution reaction, Dalton Trans. 52 (2023) 10064–10070. https://doi.org/10.1039/D3DT01469J.

[15] Y. Liu, Y. Wang, H. Wen, Y. Han, S. Deng, Green Preparation of CNTs/Graphite Supported NiFe Carbonate Hydroxides for Oxygen Evolution Reaction, ChemCatChem 14 (2022) e202200453. https://doi.org/10.1002/cctc.202200453.

[16] J. Zhang, L. Hao, Z. Chen, Y. Gao, H. Wang, Y. Zhang, Facile synthesis of Co–Fe layered double hydroxide nanosheets wrapped on Ni-doped nanoporous carbon nanorods for oxygen evolution reaction, Journal of Colloid and Interface Science 650 (2023) 816–824. https://doi.org/10.1016/j.jcis.2023.06.199.

[17] H. Li, Y. Wang, C. Liu, S. Zhang, H. Zhang, Z. Zhu, Enhanced OER performance of NiFeB amorphous alloys by surface self-reconstruction, International Journal of Hydrogen Energy 47 (2022) 20718–20728. https://doi.org/10.1016/j.ijhydene.2022.04.200.

[18] N.A. Khan, I. Ahmad, N. Rashid, S. Hussain, R. Zairov, M. Alsaiari, A.S. Alkorbi, Z. Ullah, Hafiz urRehman, M.F. Nazar, Effective CuO/CuS heterostructures catalyst for OER performances, International Journal of Hydrogen Energy 48 (2023) 31142–31151. https://doi.org/10.1016/j.ijhydene.2023.04.308.

[19] M. Dai, H. Fan, G. Xu, M. Wang, S. Zhang, L. Lu, Y. Zhang, Boosting electrocatalytic oxygen evolution using ultrathin carbon protected iron–cobalt carbonate hydroxide nanoneedle arrays, Journal of Power Sources 450 (2020) 227639. https://doi.org/10.1016/j.jpowsour.2019.227639.

[20] Y. Li, J. Chen, P. Cai, Z. Wen, An electrochemically neutralized energy-assisted low-cost acid-alkaline electrolyzer for energy-saving electrolysis hydrogen generation, J. Mater. Chem. A 6 (2018) 4948–4954. https://doi.org/10.1039/C7TA10374C.
